# Survival, Virulent Characteristics, and Transcriptomic Analyses of the Pathogenic *Vibrio anguillarum* Under Starvation Stress

**DOI:** 10.3389/fcimb.2018.00389

**Published:** 2018-11-16

**Authors:** Xiaojian Gao, Daming Pi, Nan Chen, Xixi Li, Xiaodan Liu, Hui Yang, Wanhong Wei, Xiaojun Zhang

**Affiliations:** College of Animal Science and Technology, Yangzhou University, Yangzhou, China

**Keywords:** *Vibrio anguillarum*, starvation, survival, virulent characteristics, transcriptome sequencing

## Abstract

Many bacteria have developed strategies for metamorphosis into sophisticated survival forms to survive extended periods of environmental stress. As a global causative agent of vibriosis in marine fish farming, *Vibrio anguillarum* (*V. anguillarum*) can efficiently grow and proliferate under environmental stress, but the specific mechanism is not clear. In the present study, survival, virulent characteristics, and transcriptomic analysis of the *V. anguillarum* BH1 were performed under starvation stress. The results demonstrated that *V. anguillarum* was still culturable and showed rippled surface after 6 months of starvation. Starved cells maintained their infectivity in half-smooth tongue sole (*Cynoglossus semilaevi*). Detection of virulence factors and virulence-associated genes in starved cells showed that the starved strain still produced β-hemolysis on rabbit blood agar, caseinase, dnase, and gelatinase, and possessed *emp*A, *vah*1, *vah*2, *vah*3, *vah*4, *vah*5, *rtx*A, *fla*A, *fla*D, *fla*E, *vir*C, *ton*B, *mre*B, *tox*R, *rpo*S, and *fts*Z virulence-related genes. In addition, we first reported the RNA-seq study for *V. anguillarum* with and without starvation treatment for a period of 6 months and emphasized the regulation of gene expression at the whole transcriptional level. It indicated that *V. anguillarum* expressed 3,089 and 3,072 genes in the control group and starvation stress group, respectively. The differently expressed genes (DEGs) of the starved strain were thereby identified, including 251 up-regulated genes and 272 down-regulated genes in comparison with the non-starved strain. Gene Ontology (GO) analysis and Kyto Encyclopedia Genes and Genomes (KEGG) enrichment analysis of DEGs were also analyzed. GO functional classification revealed that among the significantly regulated genes with known function categories, more genes affiliated with signal transducer activity, molecular transducer activity, and cell communication were significantly up-regulated, and more genes affiliated with cellular macromolecule, cellular component, and structural molecule activity were significantly down-regulated. In addition, the DEGs involved in the pathway of two-component system was significantly up-regulated, and the pathways of ribosome and flagellar assembly were significantly down-regulated. This study provides valuable insight into the survival strategies of *V. anguillarum* and suggests that a portion of the bacterial populations may remain pathogenic while persisting under starvation stress by up-regulating or down-regulating a series of genes.

## Introduction

*Vibrio anguillarum* is a gram-negative bacterium causing vibriosis in fish and shellfish species, and is widely distributed in marine and estuarine environments around the world (Toranzo et al., 2005; Kim et al., 2012; Ma et al., 2017). Fish infected by *V. anguillarum* showed typical signs of a generalized septicemia with hemorrhage on the base of fins, exophthalmia, and corneal opacity (Frans et al., 2011). It has brought enormous economic losses to aquaculture businesses due to the wide range of *V. anguillarum* infections among the aquaculture species including Atlantic salmon (*Salmo salar*), rainbow trout (*Oncorhynchus mykiss*), sea perch (*Dicentrarchus labrax*), ayu (*Plecoglossus altivelis*), Atlantic cod (G*adus morhua*), puffer fish (*Takifugu rubripes*), and manila clam (*Ruditapes philippinarum*) (Rodkhum et al., 2005; Mikkelsen et al., 2007; Higuera et al., 2013; Álvarez et al., 2016; Nie et al., 2017; Ren et al., 2017; Liu et al., 2018). Due to the great losses caused by *V. anguillarum* to the aquaculture industry, more attention has been paid extensively to the research regarding this opportunistic pathogen.

Nutrient insufficiency is one of the most common environmental stresses which bacteria routinely encounter in natural ecosystems. Similar to many other marine bacteria, *Vibrio* spp. have been observed to survive for a long period during starvation by sequential changes in cell physiology and gradual changes in morphology. The reduction in size, e.g., rod-shaped bacteria turning into coccoid, is beneficial to minimize the requirements for cell maintenance and adapt to environmental stresses (Chen et al., 2009). Many reports have demonstrated that *V. cholerae, V. vulnificus, V. parahaemolyticus*, and *V. alginolyticus* could enter into a viable but non-culturable stage when exposed to a low-nutrient environment (Jiang and Chai, 1996; Mcdougald et al., 1998; Chaiyanan et al., 2007; Amel et al., 2008; Abdallah et al., 2010; Su et al., 2013). Besides, under such changing environmental conditions, the metabolic pathways of bacteria would be altered to maintain their cellular functions. *Vibrio anguillarum* could persist under conditions of carbon starvation for a long period (Fujiwara-Nagata and Eguchi, 2004). Eguchi et al. reported that starvation of infective *V. anguillarum* induced a reduction in growth and pathogenicity and led to a change in shape from a short rod to a spherical microcell (Eguchi et al., 2000). Larsen et al. observed that *V. anguillarum* was chemotactic to serine in the temperature range 5–25°C and in 0.8–2.7% NaCl, and also showed a high chemotactic response after starvation (Larsen et al., 2004)*. Vibrio anguillarum* may also develop bile salt resistance through biofilm formation while persisting within intestinal environment (Wang et al., 2003). In addition, some studies have demonstrated that environmental stress could affect the expression of virulence genes in *V. anguillarum* that have implications on the competitiveness, stress tolerance, and the ability of *V. anguillarum* to cause infection (Crisafi et al., 2014). Therefore, it is important to study the gene expression of microorganisms in order to understand the pathogenicity and molecular adaptations of microorganisms under environmental stress.

In this study, we focused on the changes of the morphology, survival, and virulent characteristics of *V. anguillarum* BH1 induced by starvation. The full transcriptome of *V. anguillarum* BH1 strain during starvation stress using high-throughput sequencing was also analyzed. The present study will provide foundations to have a better understanding of virulence and survival mechanism of the bacterium under extreme environmental conditions.

## Materials and methods

### Bacterial strains and starvation stress

*V. anguillarum* strain BH1 was originally isolated in our lab from diseased half-smooth tongue sole (*C. semilaevi*) which caused mass mortalities of cultured half-smooth tongue sole in a farm in Ganyu County of Jiangsu Province, China. It was stored in 2216E marine broth supplemented with 10% glycerol frozen at −80°C in our lab, and routinely cultured on 2216E marine agar at 28°C for 16 h. The bacterial colony from the strain was inoculated into 100 mL of 2216E marine broth and incubated at 28°C for 16 h while being shaken. *Vibrio anguillarum* cells were collected by centrifugation at 8,000 × g for 10 min at 4°C and washed twice with sterile saline solution (0.9% w/v NaCl). The cells were then inoculated into sterile Erlenmeyer glass flasks (300 mL) containing 100 mL of sterile saline to a concentration of ~10^9^ CFU/mL. Three repetitions of microcosms were incubated in a static state at 28°C as starvation treatment group, i.e., without any nutrient supplement added periodically, and monitored for a period of 6 months. The freshly prepared log-phase *V. anguillarum* cell culture without starvation treatment was used as the control.

### Ultrastructural analysis

Morphological changes between 6-months starved and non-starved cells were observed under a scanning electronic microscope (SEM) as previously described with minor modification (Arias et al., 2012). Briefly, 10 μL of bacteria was fixed in 2.5% glutaraldehyde (v/v) on 8 × 8 mm glass slides at 4°C overnight. Then, samples were dehydrated in a graded ethanol series (50, 70, 90, and 100%), coated with gold palladium alloy in an EMS 550X (Electron Microscopy Science), and examined under Zeiss EVO 50 electronic microscope (Zeiss, Germany).

### Viability measure

The viability of *V. anguillarum* was measured at 1, 2, 3, 4, and 6 months post-inoculation. 100 μL of samples were serially 10-fold diluted in sterilized distilled water and were plated on LB agar in triplicate. The number of CFU were expressed as the mean ± standard deviation (SD).

### Bacterial motility test

The motility of *V. anguillarum* BH1 was measured after 0, 1, 2, 3, 4, 5, and 6 months starvation. Bacterial motility testing was performed as described previously (Xu et al., 2014). Briefly, the *V. anguillarum* cells in TSB, which had reached the early stationary phase, were separately spread on TSA supplemented with 1.5% agar. The plates were incubated at 28°C until formation of the colonies. A single colony from plates supplemented with 1.5% agar was inoculated by a puncture in the middle of the plates containing 0.4% agar. The diameter of the halo surrounding the punctured portion of the agar media was measured 24 h post-inoculation.

### Determination of extracellular enzymes and hemolysin of starved cells

The presence of caseinase, gelatinase, lipase, lecithinase, dnases, and hemolysin of the 6-months starved cells were determined by directly streaking the starved cells onto 2216E nutrient agar that contained 8% gelatin (gelatinase test), 1% Tween-80 (lipase test), 10% skim milk (caseinase test), 1% DNA with 0.005% methyl green (dnases test), and 10% egg yolk (lecithinase test) as substrate. Besides, these strains were tested for β-hemolytic activity on nutrient agar supplemented with 7% rabbit erythrocytes. These plates were incubated for 24–48 h at 28°C, and the presence of the lytic halo around the colonies was observed.

### Virulence-related genes assay of starved cells

The 6-months starved *V. anguillarum* was subjected to PCR assays to detect the virulence-related genes, including structural genes of the flagellum (*flaA, flaD, flaE*), the haemolysin genes (*vah*1, *vah*2, *vah*3, *vah*4, and *vah*5), the virulence regulator gene (*toxR*), the cell surface components genes (*vir*A, *vir*B, and *virC*), zinc metalloprotease gene (*emp*A), trans-acting transcriptional activator (*angM, angR*) repeat in toxin gene (*rtx*A), iron vibriocin transport gene (*tonB*), cell division protein gene (*ftsZ*), rod shaping protein gene B subunit (*mreB*), and regulation of specific gene expression gene (*rpoS*). The specific primers used are shown in Table 1. The following components were added into the mix to obtain 25 μL of PCR mixture: 12.5 μL of 2 × Easy Taq PCR Super® Mix (TRANS), 0.5 μL of each of the primers (10 μM), 1 μL of DNA template, and 10.5 μL of ddH_2_O. The thermal cycling protocol used included initial denaturation at 94°C for 5 min, followed by 30 cycles of denaturation at 94°C for 1 min, annealing at 52–60°C for 30 s, and extension at 72°C for 1 min. A final extension step of 72°C for 7 min was also applied.

**Table 1:** Sequence of primers used for detection of virulence genes.

**Target gene**	**Product size (bp)**	**PCR primers sequence (5^′^-3^′^)**	**References**
*empA*	248	F: CCTTTAACCAAGTGGGCGTA	Chen et al., 2005
		R: CGATTTGTAAGGGCGACAAT	
*vah1*	493	F: TGCGCTATATTGTCGATTTCAGTT	Rodkhum et al., 2006
		R: GCACCCGTTGTATCATCATCTAAG	
*vah2*	876	F: ATGAACGAAGATAACCCCCAGA	GenBank AB189395
		R: TCACTCTTCTGCTATCACTGG	
*vah3*	1128	F: ATGACTTCTTCTAAATTTTCGTTATGTGCG	GenBank AB189396
		R: GATAGAGCGGACTTTGCTTG	
*vah4*	603	F: ATGAAAACCATACGCTCAGCATCT	Rodkhum et al., 2006
		R: TCACGCTTGTTTTTGGTTTAAATGAAATCG	
*vah5*	1758	F: ATGCTCACGATAAGCCCTTTTAGAT	GenBank AB189398
		R: TCAAGGGTTAGGCGCGTGAT	
*rtxA*	441	F: GCCTTCTTCGCCTAAACCT	GenBank EU155486
		R: ATTCGCAGCCACTACCAG	
*flaA*	435	F: GTGCTGATGACTTCCGTATGG	Lu, 2010
		R: GCTCTGCCCGTTGTGAATC	
*flaD*	425	F: TGACAGCACAGCGTTACCT	McGee et al., 1996
		R: GTTATCCGCACCGATTTG	
*flaE*	431	F: CAGCCTGCTTCAGCGTAT	McGee et al., 1996
		R: TTTGCCCATTGATGTAGGT	
*virC*	344	F: TCCTTCCTTGTGGTTAGCATTG	Lu, 2010
		R: GCCTCCGCAATAATCCAGTC	
*tonB*	195	F: GGCGTAGAAGGTTTCGTT	GenBank AY644719
		R: CTCCACAGTCACGGTTTG	
*mreB*	711	F: GCGTGATTGCGGATTTC	GenBank DQ907406
		R: CGACTGGTATTCCCGTTTC	
*toxR*	397	F: AACACCACCAACGAGCCT	GenBank AB042547
		R: GACCACCAGTCGCAATCA	
*rpoS*	492	F: CAAAGCGATGACGATG	GenBank AY695434
		R: TTCTTCTGCGGTAGGTTC	
*ftsZ*	185	F: ATTTGCGAGTGCGAATGA	GenBank DQ907334
		R: CCATCTCTGCCGCTTCT	
*angM*	453	F: TGAAGTTGAGCCTCGTAA	Lu, 2010
		R: TCAGACCTGTTGATTCGT	
*angR*	957	F: AAGAGTGAGCCAATGCGTAG	Chen et al., 1996
		R: CTCCGAATCCATAACGATGA	
*virA*	314	F: TCAGAGAGGATTGATAGGT	Lu, 2010
		R: ACACTTATGGGATGTAACAC	
*virB*	496	F: GTAGAACGGCAGATTGGT	Lu, 2010
		R: GAGCCATAGCGATAGATTG	

### Bacterial virulence assay

The pathogenicity of 6-months starved and non-starved *V. anguillarum* was tested in healthy half-smooth tongue sole raised in aquaria containing 80 L of seawater supplemented with oxygen. The starved and non-starved *V. anguillarum* cells were streaked onto 2216E nutrient agar plates, and incubated at 28°C for 24 h. Then purified 6-months starved and non-starved *V. anguillarum* was cultured at 28°C for 24 h in 2216E liquid medium. Twenty fish (average 8–10 cm in length) in each group were injected intraperitoneally with 0.1 mL live cells with bacteria ranging from 10^4^ to 10^7^ CFU/mL. The control fish were inoculated with 0.1 mL sterile saline solution. Inoculated fish were cultured at 25°C and observed for 7 days. Average values were taken for calculating percentage of mortality, and the median lethal dose (*LD*_50_) values were calculated as described by Behreans and Karber (1953). All experiments were repeated three times. All the treatments involving animals were strictly in accordance with the guidelines of Animal Experiment Ethics Committee of Yangzhou University.

### RNA extraction, library construction, and illumina sequencing

Total RNA was extracted from 6-months starved and negative control group cells using the EasyPure RNA kit (TransGen Biotech, Beijing, China) according to the manufacturer's instructions. The integrity and size distribution of the dissolved RNA samples was checked with Agilent 2100 Bioanalyzer (Agilent technologies, USA) before storage at −80°C. The cDNA was reverse transcribed from these RNA samples and then quantified using several kits according to the manufacturer's instructions as previously reported. Briefly, mRNA was purified from total RNA using poly-T oligo-attached magnetic beads. The rRNA was removed using a kit that leaves behind the mRNA. All mRNA was broken into short fragments by adding a fragmentation buffer. Considering these short fragments random hexamer-primer was used to synthesize the first-strand cDNA. The second-strand cDNA was synthesized using a buffer, dATPs, dGTPs, dCTPs, dUTPs, RNase H, and DNA polymerase I after removing dNTPs. The purified fragments were further subjected to the addition of poly (A) addition and end reparation. Afterwards, the short fragments were connected with sequencing adapters and the UNG enzyme was used to degrade the second-strand cDNA. Then the product was purified by MiniElute PCR Purification Kit before PCR amplification. The library was sequenced on the Illumina Hiseq2500 platform at Novogene Co., Ltd. (Beijing, China).

### Transcriptome analysis

The clean reads of starved and non-starved samples were acquired from the raw data by removing the adaptors, poly-N, and low-quality reads. Next, we compared the clean reads with the reference genome and sequence using Bowtie2. Gene coverage, i.e., ratio of observed reads to the number of total nt bases, was also checked during the clean read comparison. The false discovery rate (FDR) was set at 0.0001 to determine the threshold of the *P*-value in multiple tests. Calculation of expression was applied by FPKM (Fragments Per kb per Million reads) method (Mortazavi et al., 2008). The resulting *P*-values were adjusted using the Benjamini and Hochberg's approach for controlling the false discovery rate. Genes that were differentially expressed between the starved and non-starved samples were determined by the DESeq R package, with a *q*-value < 0.05 and absolute value of log2-fold change >1 set as the threshold for significance of gene differential expression. In addition, Gene Ontology (GO) analysis and Kyto Encyclopedia Genes and Genomes (KEGG) enrichment analysis of DEGs were used to annotate and classify the differentially expressed genes (Conesa et al., 2005; Kanehisa et al., 2014).

### Validation of genes using qRT-PCR

Eight genes including *vah* (hemolysin), *toxR* (transcriptional regulator), *LuxR* (LuxR family transcriptional regulator), *CheY* (chemotaxis protein), *flgA, flgC, flgD* (flagellar protein), and *fliD* (flagellar cap protein) were selected for the confirmation of RNA-seq data by Quantitative real-time PCR (qRT-PCR). The RNA samples were extracted using an EasyPure RNA kit (TransGen Biotech, Beijing, China), according to the manufacturer's instructions. The cDNAs were then synthesized using a TransScript One-Step gDNA Removal and cDNA Synthesis Supermix (TransGen Biotech, Beijing, China), as recommended by the manufacturer. 16S rRNA was used as the internal control. The primers used for qRT-PCR are shown in Table 2. Real-time PCR was performed using the Roche LightCycler® 96 System with a TransStart Top Green qPCR SuperMix following the instructions of the manufacturer. The relative gene expressions were determined using 2^−ΔΔ*Ct*^ method. The expression of the detected genes was analyzed by One-Way ANOVA using SPSS 16.0. All quantitative data were expressed as means ± SD.

**Table 2:** Primers used for the detection of DEGs by qRT-PCR.

**Target gene**	**PCR primers sequence (5^′^-3^′^)**
*vah*	F: ATTGTCTGGCGGTGAAAG
*toxR*	R: GGTGCTGCACATCTAACG
	F: ATCTAACCGATGACTACTTGA
	R: TCGTTGATGACCCTGACT
*CheY*	F: ACAGCCACTTGAGTCTACGA
	R: TTATTGCGCCACTTTCCA
*LuxR*	F: ATGTGGATTTGCTGCTGT
	F: AGGCGATTTGTTTGTTGA
*flaA*	F: TGCGGCAGGCTTACAAAT
	R: CCTTGTCCTTTCCTTGCTCTGCAAG
*flaC*	F:CGGCAAATGGACAAGATA
	R:CACCCAGTTGAGCACGAT
*flaD*	F: GACAGCACAGCGTTACCT
	R: TCATTCATCGCACCCTCA
*fliD*	F: GCTCAAAGAGTCGCTGGAT
	R: TGATTACCGAAGCACGAA

## Results

### Phenotype changes under starvation stress

**Figure 1:**
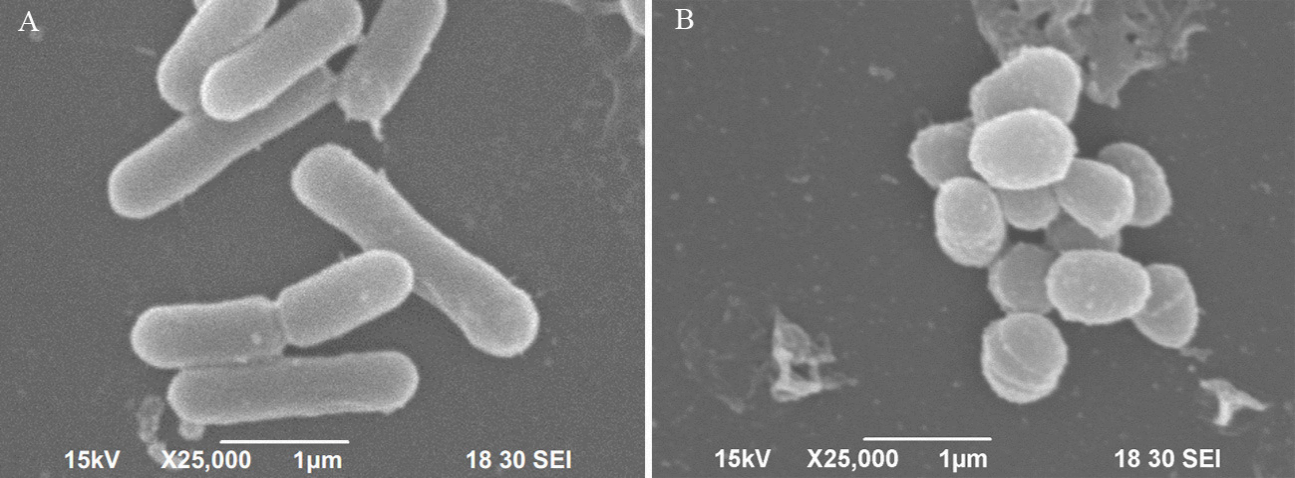
Morphological changes of *V. anguillarum* cells after starvation revealed by scanning electronic microscopy. **(A)** Initial cells, **(B)** 6-months starved cells.

**Figure 2:**
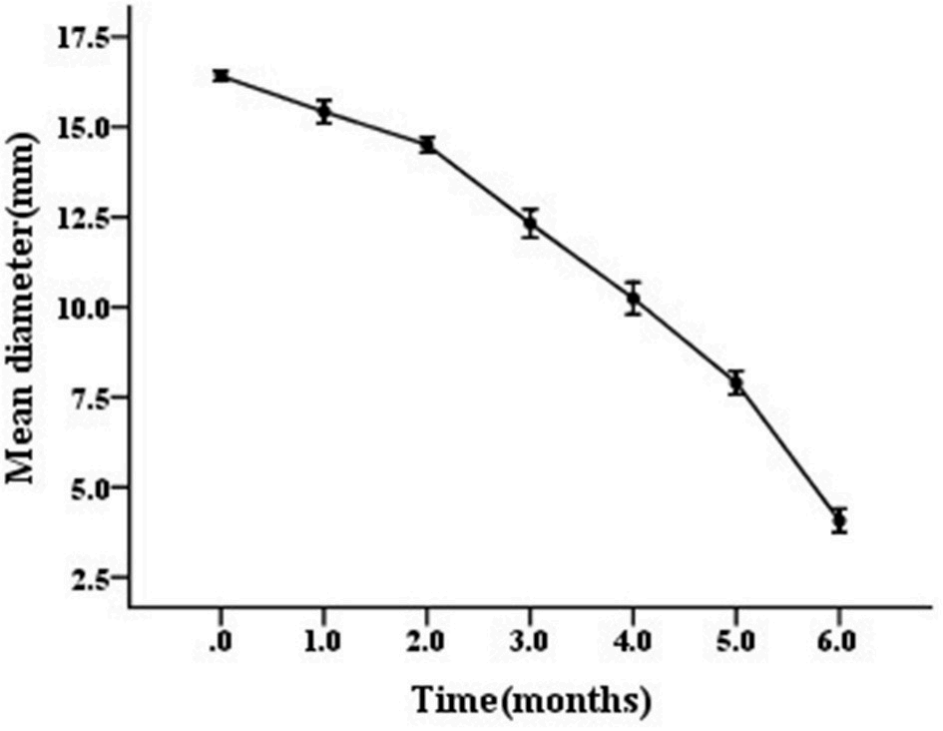
Motility of starved *V. anguillarum* BH1 during 6-months starvation at 28°C. Error bars: ±SD.

The scanning electron micrograph revealed that the non-starved *V. anguillarum* were mainly short rods, and changed to spheres after the 6-months starvation. After 6-months starvation, the average cell length of the initial *V. anguillarum* had significantly decreased from 2.0 ± 0.2 μm to 1.0 ± 0.2 μm (Figure 1). Additionally, the starved cells showed a rippled surface, much rougher than that of the non-starved cells. Furthermore, the motility of *V. anguillarum* was determined by measuring the diameter of the halo on the agar plate. The results in Figure 2 showed that the motility of *V. anguillarum* decreased after starvation. In addition, the phenotypic determination of putative virulence factors showed that the 6-months starved cells could still produce β-hemolysis on rabbit blood agar, caseinase, dnase, and gelatinase.

### Survival of the starved *V. anguillarum*

**Figure 3:**
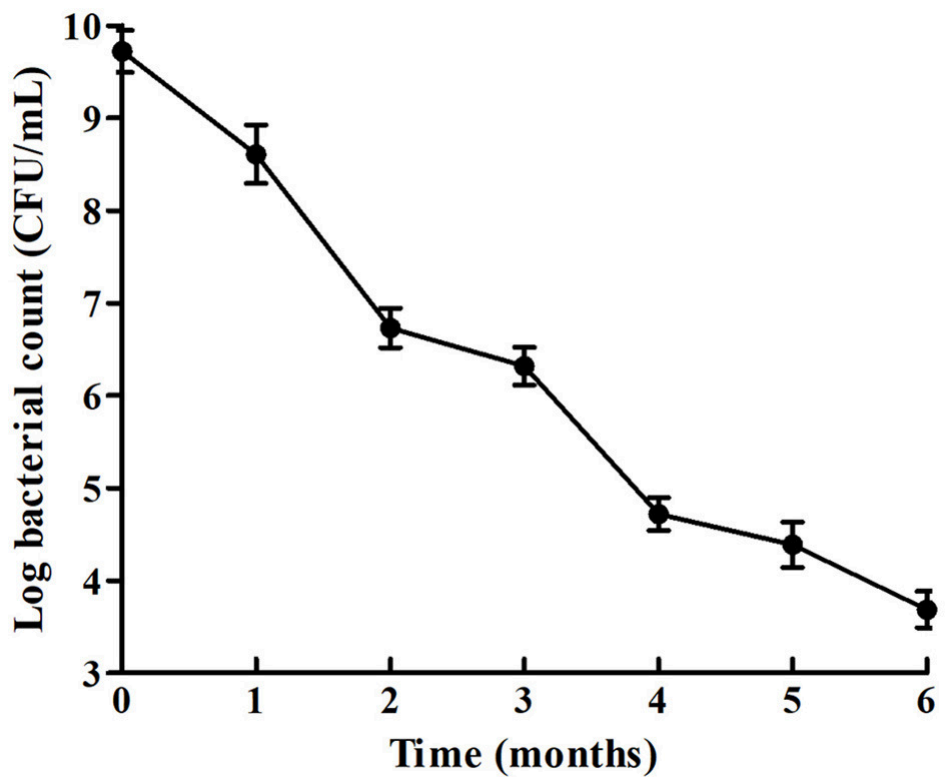
Survival curves of *V. anguillarum* BH1 under starvation stress. Error bars: ±SD.

The survival rate of *V. anguillarum* BH1 under starvation at 28°C was determined over a 6-months period. The cell enumeration of *V. anguillarum* BH1 is shown in Figure 3. Overall, *V. anguillarum* BH1 was still culturable after 6 months of starvation. The cell counts were on the decline during the 6-months starvation period with Log10 CFU/mL changing from 9.7224 ± 0.2254 to 3.6877 ± 0.1966.

### Virulence related genes of starved cells

**Figure 4:**
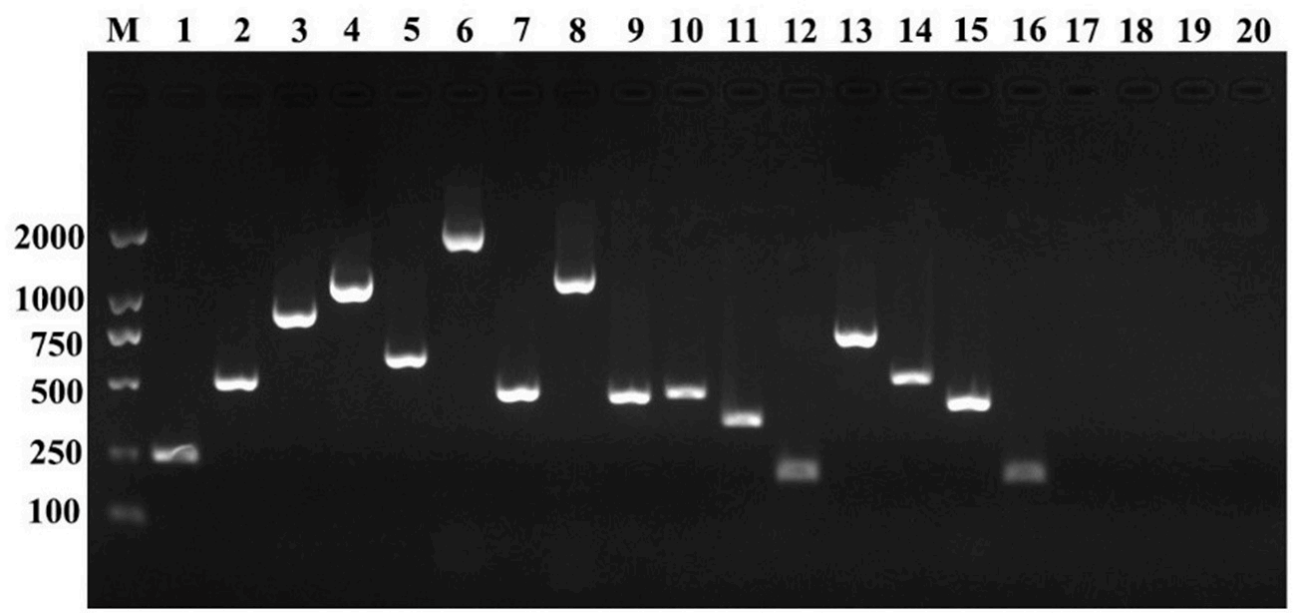
Detection of virulence-related genes of the initial *V. anguillarum* cells. M, DL 2000; lane 1, *emp*A; lane 2, *vah*1; lane 3, *vah*2; lane 4, *vah*3; lane 5, *vah*4; lane 6, *vah*5; lane 7, *rtx*A; lane8, *fla*A; lane 9, *fla*D; lane 10, *fla*E; lane 11, *vir*C; lane 12, *ton*B; lane 13, *mre*B; lane 14, *tox*R; lane 15, *rpo*S; lane 16, *fts*Z; lane 17, *ang*M; lane 18, *ang*R; lane 19, *vir*A; lane 20, *vir*B.

The PCR profiles of the amplified virulence**-**related genes from the starved cells are presented in Figure 4. The results showed that *emp*A, *vah*1, *vah*2, *vah*3, *vah*4, *vah*5, *rtx*A, *fla*A, *fla*D, *fla*E, *vir*C, *ton*B, *mre*B, *tox*R, *rpo*S, and *fts*Z genes were detected in the starved cells. However, *ang*M, *ang*R, *vir*A, and *vir*B genes were not detected using the given primers in this study.

### Pathogenicity of starved *V. anguillarum*

The non-starved and 6-months starved *V. anguillarum* were used to infect a half-smooth tongue sole. The results showed that the *V. anguillarum* starved for 6 months were able to infect a half-smooth tongue sole by intraperitoneal injection. As shown in Table 3, the starved cells presented an overall decline of virulence compared to that of the non-starved cells. The *LD*_50_ value of non-starved and 6-months starved was 1.747 × 10^6^ CFU/mL and 7.357 × 10^6^ CFU/mL for half-smooth tongue sole, respectively.

**Table 3:** Pathogenicity of non-starved and 6-months starved *V. anguillarum* to half-smooth tongue sole.

**Bacteria**	**Fish amount**	**Bacterial concentration (CFU/mL)**	**Number of dead fish during infection (day)**								**Mortality (%)**
			**1**	**2**	**3**	**4**	**5**	**6**	**7**	**Total**	
Non-starved cells	20	3.6 × 10^7^	0	9	8	3	0	0	0	20	100.0
	20	3.6 × 10^6^	0	4	6	0	1	0	0	11	55.0
	20	3.6 × 10^5^	0	2	3	0	0	0	0	5	25.0
	20	3.6 × 10^4^	0	0	0	0	0	0	0	0	0.0
Starved cells	20	3.2 × 10^7^	0	3	7	4	1	0	0	15	75.0
	20	3.2 × 10^6^	0	0	3	3	0	0	0	6	30.0
	20	3.2 × 10^5^	0	0	0	3	0	0	0	3	15.0
	20	3.2 × 10^4^	0	0	0	0	0	0	0	0	0.0
Control	20	0	0	0	0	0	0	0	0	0	0.0

*Data are presented as average value of three repeats at each concentration*.

### Sequencing quality assessment and gene expression annotation

To identify the molecular mechanisms involved in response to starvation, cDNA samples of *V. anguillarum* strain BH1 with and without starvation treatment were sequenced using Illumina HiSeq™ 2500 system. After removing adaptors, poly-N, and low-quality reads, a total of 16,430,586 clean reads for control group cells and 14,126,118 reads for starved cells were obtained. Proportions of clean reads mapped back to genome and genes can provide an overall assessment of the sequencing. After the alignment of the sequence reads with the reference genome sequence of *V. anguillarium*, a total of 93.24 and 93.81% clean reads could be mapped in control and starved groups, respectively (Table 4). Only those reads entirely aligning within exonic regions matched (reads from exon-exon junction regions did not match). In addition, the sequencing results indicated that *V. anguillarum* strain BH1 expressed 3,089 and 3,072 annotated genes before and after starvation stress (Table 5).

**Table 4:** Summary of the reads mapping to the reference transcriptome of the control group and the starvation stress group.

**Item**	**Control group (Mapping to Genome)**	**Starvation stress group (Mapping to Genome)**
Total reads	16,430,586	14,126,118
Total mapped	15,320,669 (93.24%)	113,251,878 (93.81%)
Multiple mapped	794,433 (4.84%)	904,200 (6.4%)
Uniquely mapped	14,526,236 (88.41%)	12,347,678 (87.41%)

**Table 5:** The gene coverage of RNA-seq of *V. anguillarum* strain BH1 before and after starvation treatment.

**FPKM interval**	**Control group**	**Starvation stress group**
0–1	616 (16.63%)	633 (17.09%)
1–3	36 (0.97%)	40 (1.08%)
3–15	217 (5.86%)	224 (6.05%)
15–60	659 (17.79%)	662 (17.87%)
>60	2,177 (58.76%)	2,146 (57.92%)
Total number of expressed genes	3,089 (83.87%)	3,072 (82.91%)

*FPKM interval >1: gene expression*.

### GO analysis of differently expressed genes

**Figure 5:**
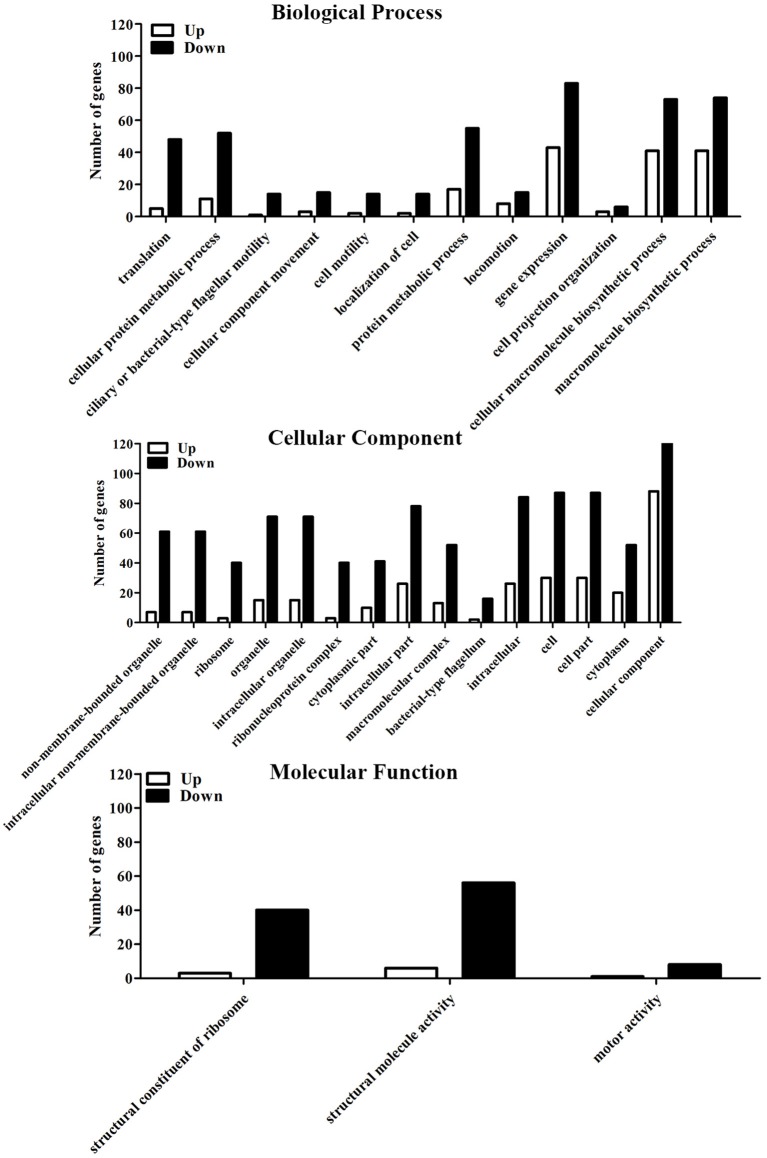
GO analysis of differential expression genes to the initial and the 6-months starved *V. anguillarum*.

Compared to the untreated group, a total of 523 differently expressed genes (DEGs) were identified in the 6-months starved group, including 251 up-regulated genes and 272 down-regulated genes. This means that 17.0% of the total number of gene expression levels had significantly changed after starvation for 6 months. GO analysis aims to annotate genes and gene products, and assimilate and disseminate annotation data via enrichment analysis. The differently expressed genes of *V. anguillarum* after nutritional stress were enriched by GO enrichment analysis and classified into three major categories: biological process, cellular component, and molecular function (Figure 5). In the biological process category, the top three categories were gene expression, cellular macromolecule biosynthetic process, and macromolecule biosynthetic process. In the cellular component category, the most abundant categories of the differential expression genes were cellular component, cell, and cell part. In the molecular function category, two of the most enriched terms were structural molecule activity and structural constituent of ribosome.

### KEGG enrichment analysis of differently expressed genes

**Figure 6:**
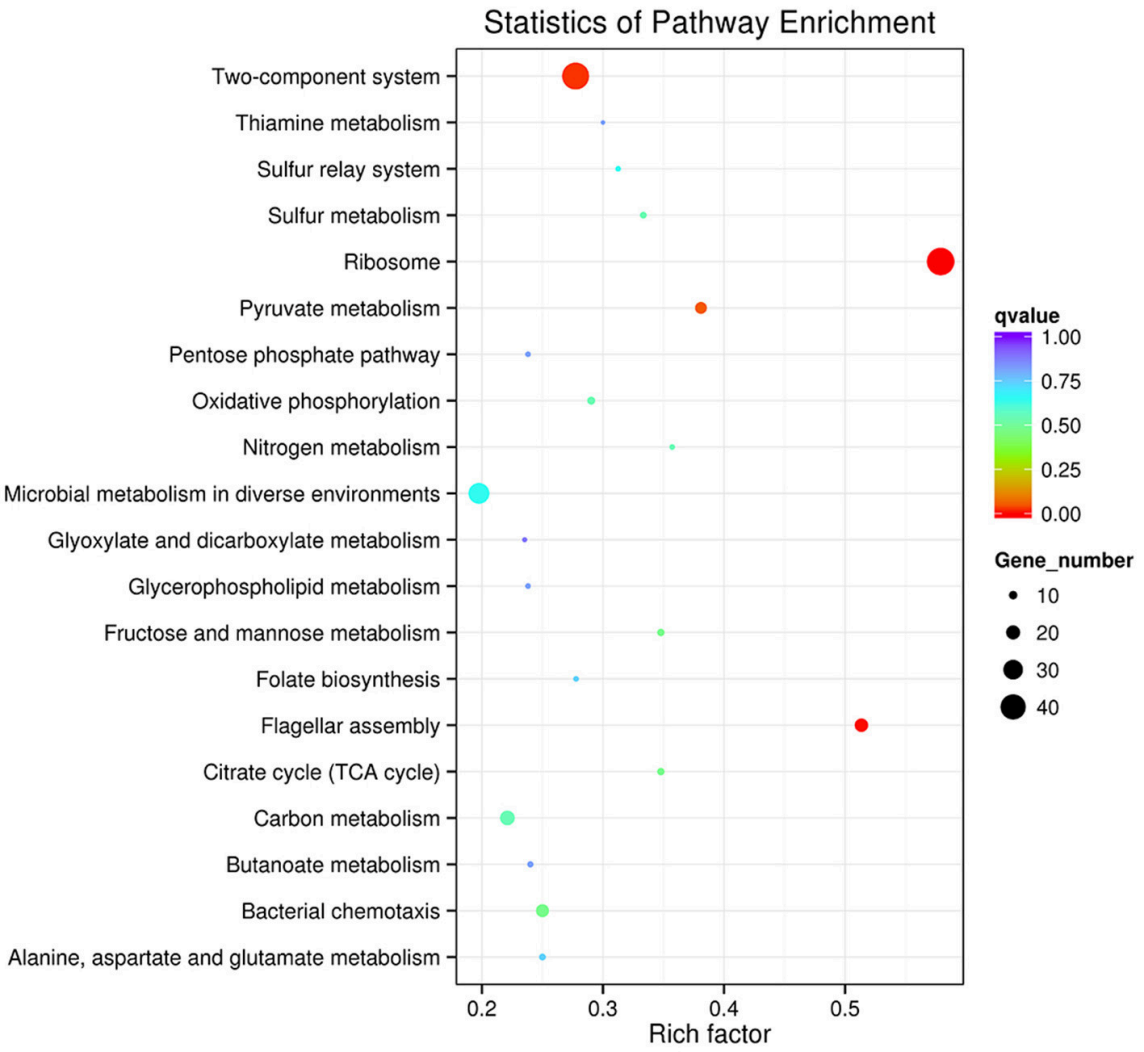
The Top20 pathways with the most significant *p*-value. The x-axis indicates percentages of DEGs belonging to the corresponding pathway. The left *y*-axis represents the top 20 pathways. The sizes of the bubbles represent the number of DEGs in the corresponding pathway, and the colors of the bubbles represent the enrichment *p*-value of the corresponding pathway. KEGG terms with *P*-value < 0.05 were considered significantly enriched by differential expressed genes.

Genes that interact with each other play an important role in generating a response to environmental stress. To perform functional classification and pathway assignment of genes after starvation stress in *V. anguillarum*, all DEGs were analyzed based on the KEGG database. KEGG enrichment analysis of differentially expressed genes revealed that the DEGs clustered to 122 biochemical metabolism and signal pathways. The most abundant pathways were fructose and mannose metabolism, citrate cycle (TCA cycle), and pyruvate metabolism. However, all *p*-values of the three pathways were >0.05, indicating that these metabolic pathways were not significantly influenced during starvation. Figure 6 shows that these transcripts, mainly belonging to ribosome, flagellar assembly, and two-component system, were significantly (*p* < 0.05) induced after starvation.

### Verification of the DEGs by qRT-PCR

**Figure 7:**
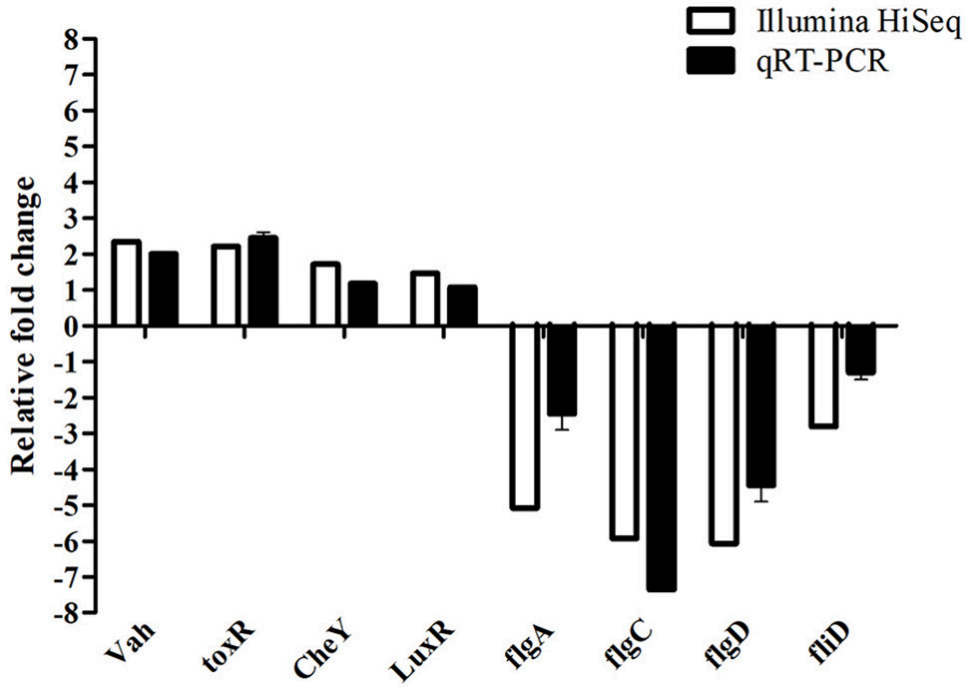
Comparative analysis of qRT-PCR and transcriptome sequencing result of differentially expressed genes of *V. anguillarum* before and after starvation stress.

To further validate the Illumina sequencing data and the differential expression level of the DEGs in the sequencing results, eight differentially expressed genes in the transcriptome data were purposefully selected for relative quantitative real-time PCR. Among these selected genes, four genes (*Vah, toxR, LuxR*, and *CheY*) were up-regulated and the other four genes (*flgA, flgC, flgD*, and *fliD*) were down-regulated. The qRT-PCR results exhibited a similar expression tendency at transcriptome analysis, which confirmed the reliability of transcriptome sequencing results (Figure 7).

## Discussion

*Vibrio* are widespread in coastal and estuarine environments. Some species, e.g., *V. anguillarum* and *V. parahaemolyticus*, are serious pathogens of aquatic vertebrates or invertebrates, and their serotypes may be diverse (Austin, 2010; Li et al., 2010). At present, 23 O serotypes (O1–O23) within *V. anguillarum* are distinguished, each displaying a different pathogenicity and host specificity. Of these, only serotypes O1 and O2, and to a lesser extent serotype O3, are associated with vibriosis in fish (Frans et al., 2011); and at least 13 O serotypes have been identified in *V. parahaemolyticus* (Broberg et al., 2011; Xu et al., 2014). However, the responses of the different serotypes of bacteria to survival, pathogenicity, and gene expression under environment stress are not well-understood. Romalde et al. (1994) reported that serotypes of *Yersinia ruckeri* reflected similar survival dynamics under starvation stress. Eguchi et al. (2003) reported that *V. anguillarum* serotype O1 can survive in freshwater under starved and low osmotic environment and is potentially the causative agent for the vibriosis of ayu. Miyamoto and Eguchi (1997) reported that a high cell density and small amounts of divalent cations in freshwater made *V. anguillarum* serotype O1 more resistant to low osmotic stress. However, the survival strategies of *V. anguillarum* to withstand different kinds of environmental stress in the aquatic environment are not well-understood. In this study, *V. anguillarum* strain BH1 was originally isolated from diseased half-smooth tongue sole, which caused mass mortalities, and we focused on the changes of the morphology, survival, virulent characteristics, and transcriptomic analyses of *V. anguillarum* BH1 induced by starvation.

It is well-known that lack of nutrients is the most common environmental stress which microorganisms will frequently confront in natural ecosystems (Kjelleberg et al., 1983; Wai et al., 1999; Desnues et al., 2003; Vatsos et al., 2003; González-Escalona et al., 2006; Su et al., 2015). In this study, the survival of *V. anguillarum* starved at 28°C over a 6-months period has been investigated. The cell viability revealed that *V. anguillarum* was still culturable after 6 months of starvation. Our results are consistent with previous reports that *Vibrio* spp. can survive for long periods, even several years, during starvation as culturable bacteria (Amel et al., 2008). In this study, the number of culturable bacteria declined gradually with time. For instance, some species would adopt an adaptive strategy, entering the so-called viable but non-culturable (VBNC) state, under stress conditions and wait for recovery until optimal conditions are restored (Igbinosa and Okoh, 2008; Su et al., 2013). According to Amel et al. (2008), *V. fluvialis* cells in the VBNC state could be metabolically reactivated, and their study showed that resuscitation of VBNC population was achieved after 48 h. The focus of our study was to understand the genetic modulations in *V. anguillarum* during starvation, including stress-induced morphological changes. *Vibrio anguillarum* cells showed a remarkable reduction in size, turning from rods to sphere, and the cell surface appeared rippled and much thicker after starvation. The morphological changes could be due to the nutrient deficiency. Besides, the starved bacteria showed rippled cell surface, which is likely to affect the attachment and colonization capabilities of this fish pathogen, leading to the variation in virulence potential. The changes observed in cell morphology during bacterial starvation were most likely an adaptive process to minimize the requirements for cell maintenance, and avail better protection against predation and other environmental stresses (Chaiyanan et al., 2007). The appearance of irregular rod-shaped cells has been demonstrated for VBNC *V. cholerae* and *V. parahaemolyticus* (Jiang and Chai, 1996; Chaiyanan et al., 2007; Abdallah et al., 2010).

Environmental stresses may stimulate the expression of genes encoding products which protect the cells from the stresses, participate in the repair of cellular damage and can cause diseases in the organism. In our study, motility of *V. anguillarum* was weaken after starvation. Meanwhile, the infectivity of the *V. anguillarum* had also decreased after starvation, indicating that starvation can attenuate the intensity of bacterial virulence to the examined fish (half-smooth tongue sole) but does not completely diminish its pathogenic potential. Moreover, the detection of virulence factors and virulence-related genes in starved *V. anguillarum* BH1 showed that the starved strain still produced β-hemolysis on rabbit blood agar, caseinase, dnase, and gelatinase, and possessed *emp*A, *vah*1, *vah*2, *vah*3, *vah*4, *vah*5, *rtx*A, *fla*A, *fla*D, *fla*E, *vir*C, *ton*B, *mre*B, *tox*R, *rpo*S, and *fts*Z virulence-related genes. The expression of these virulence genes in *V. anguillarum* may be affected by starvation. Crisafi et al. (2014) reported that environmental stress affects the expression of virulence genes in *V. anguillarum*, e.g., transcription levels of *empA, angR*, and *fatA* increased under conditions of 15°C and iron depletion, which have implications on the competitiveness, stress tolerance, and the ability of *V. anguillarum* to cause infection. Similarly, Saint-Ruf and Matic (2006) reported that under nutrient limitation, the high intracellular concentration of RpoS diminished nutritional competence and increased stress resistance. Knowing the expression of genes of bacteria responding to environmental stimuli is important for understanding the factors determining pathogenicity and infection, and molecular adaptations that follow a specific stress (Boor, 2006).

Bacterial pathogens are frequently exposed to a variety of stresses in their natural environment and in their host systems, which may lead to increased virulence, adaptability, and resistance (Awan et al., 2018). Previous studies mainly focused on phenotype changes and survival of *V. anguillarum* under environmental stresses (Eguchi et al., 2000, 2003; Fujiwara-Nagata and Eguchi, 2004; Larsen et al., 2004; Kim et al., 2012; Crisafi et al., 2014). However, the specific events and pathways at the transcriptomic level of *V. anguillarum* under environmental stress, especially in relation to survival and pathogenicity, has never been studied. To understand the survival ability of V. anguillarum further, performed the transcriptomic analysis of *V. anguillarum* under environmental stresses. The full transcriptome of *V. anguillarum* during starvation stress, using high-throughput sequencing, may enhance the understanding of the survival, virulence, and metabolism-related factors in environmental suitability of *V. anguillarum*.

In this study, transcriptome sequence analysis was performed to assess the variation of virulence-related and metabolism-related factors in *V. anguillarum* and its survival abilities under starvation stresses. According to this study, 251 up-regulated genes and 272 down-regulated genes were identified after exposure to starvation stress by using the RNA-Seq technology, which very likely played an important role in generating a response to starvation stress. There were 30 GO categories related to the starvation stress response of *V. anguillarum* and the key biological processes associated with starvation stress response may be a metabolic process, cell and catalytic activity, binding and transporter activity process. KEGG pathway analysis in this study has provided important information to explain the relationship between the gene function and regulation mechanism during the long-term survival of *V. anguillarum*. In addition to the gene enrichment pathways, there are many other significant pathways, such as ABC transporters, flagellum assembly, fatty acid degradation, fatty acid metabolism, alanine, aspartate and glutamate metabolism, which were also closely related to starvation stress (Higgins, 2001; Svensson et al., 2014). In-depth analysis of sequencing results displayed that the expressions of the nutrients that transport related genes were down-regulated, including oligopeptide, simple sugars, and phospholipids, after 6 months of starvation. However, the nutrient transfer related genes, such as monosaccharide transporters gene and phospholipid transfer protein gene were significantly up-regulated and likely to promote an active metabolism pathway in *V. anguillarum* under nutritional stress. On one side, the analysis of fatty acid metabolism pathway showed that the expression level of fatty acid synthesis-related genes were significantly down-regulated. On the other side, the expression level of fatty acid degradation-related genes were significantly increased. The functional impacts of the genes which were up-regulated or down-regulated under the starvation need to be elucidated in the future.

In our study, long-term starved cells of *V. anguillarum*, which showed reduction in size and changed from spherical to rod shape, were still culturable and pathogenic. The transcriptome sequence analysis proposed that *V. anguillarum* could survive under starvation stress by up-regulating or down-regulating a series of genes predominantly related to nutrient transfer and metabolism. The identified key genes could be important high-value drug targets, which provide new ways to effectively control infection of *V. anguillarum* clinically.

## Author contributions

XG analyzed the data and wrote the manuscript. XG, DP, NC, and XL conducted *V. anguillarum* under starvation stress assay, ultrastructural analysis, viability measure, swimming motility assay, determination of extracellular enzymes and hemolysin of starved cells, virulence gene assay of starved cells, pathogenicity of starved cells assay. XDL and HY conducted the transcriptomic analyses of the pathogenic *V. anguillarum* under starvation stress. WW and XZ designed the research and revised the manuscript. All authors read and gave final approval of the manuscript.

### Conflict of interest statement

The authors declare that the research was conducted in the absence of any commercial or financial relationships that could be construed as a potential conflict of interest.
